# Influence of right ventricular remodeling on ventricular function, flow and energetics in pulmonary regurgitation vs. stenosis: a 4-dimensional phase contrast MRI and admittance catheterization study

**DOI:** 10.1186/1532-429X-18-S1-O95

**Published:** 2016-01-27

**Authors:** Filip Konecny, James M Hammel, Joao Filipe Fernandes, Andreas Schuster, Quanliang Shang, John Lof, Leonid Goubergrits, Titus Kuehne, Shelby Kutty

**Affiliations:** 1 grid.266813.80000000106664105Chiildren's Hospital and Medical Center, University of Nebraska Medical Center, Omaha, NE USA; 2 Transonic Scisense, Inc, London, ON Canada; 3 grid.418209.60000000100000404German Heart Institute, Berlin, Germany; 4 Heart Centre Gottingen, Gottingen, Germany

## Background

We studied ventricular hemodynamics, flow and energy characteristics with right ventricular (RV) remodeling in pressure and volume loaded right ventricle.

## Methods

Catheterization with pressurevolume (PV) studies was performed in control pigs, and in models of pulmonary stenosis (PS) and pulmonary regurgitation (PR) at 1012 weeks of model creation. Cardiac magnetic resonance imaging with assessment of ventricular volumes, function and flow (four-dimensional phasecontrast magnetic resonace imaging, 4DPCMRI) was performed at baseline and at 10-12 weeks on the same day of catheterization. 7F admittance catheter in the RV/LV was connected to an admittance control (ADVantage, Transonic Scisense Inc.) and data at baseline and preload reduction recorded. From 4DPCMRI, phasecontrast velocities were computed over 1 cardiac cycle (25 timesteps, MevisFlow and CAIPI, Fraunhofer MEVIS), with segmentation of a moving left ventricle mask based on cine images. The segmented mask was applied to the respective calculated 3D velocity vector field per timestep. Mean RV kinetic energy (KE) and KE curve profiles were measured, normalized for RV volumes and compared between PS and PR groups. RV stroke work, stroke power and power efficiency was determined.

## Results

In 17 pigs (7PS, 7PR, 3 controls, mean weight 58 kg) studied, RV endsystolic (ESP) and enddiastolic (EDP) pressures were elevated in PS compared to PR and controls (ESP 55.7 ± 19 vs.20.6 ± 5.3 vs.13.4 ± 2; p < 0.001 and EDP 9.8 ± 2.7 vs.5.9 ± 1.7 vs. 1.4 ± 1.3; p < 0.05). The RV dp/dt min in PS vs. PR vs. control was -563 ± 271 vs.195 ± 63) (p < 0.05). PR had higher RV power efficiency compared to PS (82 ± 12 vs. 24 ± 14%) post-procedure. Both groups had similar KE pre procedure (8.5 ± 5 mJ/L in PS, 9 ± 6 mJ/L in PR) but significant changes in KE postprocedure (11.5 ± 4 mJ/L in PS, 24 ± 5 mJ/L in PR).

RV stroke volume (ml) in PS and PR were 39 ± 5.5 vs. 64.4 ± 13.4. PS showed higher slope curvilinear relationship of end systolic elastance RV Ees (2.11 vs. 0.82 in PR) during preload reduction, while the difference in EDPVR was nonsignificant. The preload recruitable stroke work linear relationship for RV showed significant difference for PS vs. PR, and vs. control (p < 0.05). The RV dp/dt max vs. EDV also showed difference between controls vs. PS and PR. RV changes influenced diastolic LV function: the EDV(ml) for PS, PR and controls were 100 ± 16, 114 ± 28 and 149 ± 22 (p < 0.05), while diastolic LV stiffness markedly rose in both PR and PS vs. controls (0.04 vs. 0.027 vs. 0.014).

## Conclusions

PS and PR have different RV work load at baseline, and differ in systolic power generated during preload reduction. RV in PR had higher KE and power efficiency compared to RV in PS. Differential power in PR and PS and ventricular interdependence likely influence LV filling, and may be responsible for altered LV stiffness.

